# Function and therapeutic potential of transient receptor potential ankyrin 1 in fibrosis

**DOI:** 10.3389/fphar.2022.1014041

**Published:** 2022-10-06

**Authors:** Yicheng Wei, Jialuo Cai, Ruiqiu Zhu, Ke Xu, Hongchang Li, Jianxin Li

**Affiliations:** ^1^ Third Affiliated Hospital of Shanghai University/Wenzhou People’s Hospital, Wenzhou, China; ^2^ Shanghai Putuo Central School of Clinical Medicine, Anhui Medical University, Hefei, Anhui, China; ^3^ Interventional Cancer Institute of Chinese Integrative Medicine, Putuo Hospital, Shanghai University of Traditional Chinese Medicine, Shanghai, China; ^4^ Shanghai Frontiers Science Center of Optogenetic Techniques for Cell Metabolism, Shanghai Key Laboratory of New Drug Design, School of Pharmacy, East China University of Science and Technology, Shanghai, China; ^5^ Musculoskeletal Organoid Research Center, Institute of Translational Medicine, Shanghai University, Shanghai, China; ^6^ Wenzhou Institute of Shanghai University, Wenzhou, China; ^7^ Department of General Surgery, Institute of Fudan–Minhang Academic Health System, Minhang Hospital, Fudan University, Shanghai, China

**Keywords:** TRPA1, fibrosis, TGF-β, inflammation, TRPA1 antagonists

## Abstract

The transient receptor potential (TRP) protein superfamily is a special group of cation channels expressed in different cell types and signaling pathways. In this review, we focus on TRPA1 (transient receptor potential ankyrin 1), an ion channel in this family that exists in the cell membrane and shows a different function from other TRP channels. TRPA1 usually has a special activation effect that can induce cation ions, especially calcium ions, to flow into activated cells. In this paper, we review the role of TRPA1 in fibroblasts. To clarify the relationship between fibroblasts and TRPA1, we have also paid special attention to the interactions between TRPA1 and inflammatory factors leading to fibroblast activation. TRPA1 has different functions in the fibrosis process in different organs, and there have also been interesting discussions of the mechanism of TRPA1 in fibroblasts. Therefore, this review aims to describe the function of TRP channels in controlling fibrosis through fibroblasts in different organ inflammatory and immune-mediated diseases. We attempt to prove that TRPA1 is a target for fibrosis. In fact, some clinical trials have already proven that TRPA1 is a potential adjuvant therapy for treating fibrosis.

## Introduction

Transient receptor potential channels are usually receptors composed of TRP-related proteins that can mediate sensory transduction and physiological responses. They make different responses to the stimuli that are involved in activation of the channels (Earley and Brayden, 2015). From the literature, we found that 28 genes, including six subfamilies, constitute the TRP superfamily. Although the six subfamilies have similar structures, their physiological functions are different and characteristic. These include TRPCs 1-7, TRPV1-6, TRPM1-8, TRPA1, TRPML1-3 and TRPP1-2. TRP channel proteins mainly allow passage of sodium ions and have medium to high permeability to divalent cations (except TRPM4 and TRPM5) in the human body. At the same time, some channels also allow metal ions and other rare divalent cations to pass through and permeate, among which calcium ions have a significant impact on human physiological functions (Watanabe et al., 2008; Bouron et al., 2015).

Transient receptor potential ankyrin 1 is the only member of the ankyrin domain family of TRP channels. TRPA1, which is highly expressed in nerve tissues, plays a key role in pain models and neurogenic inflammation and can be activated by stimulating signals or stimulating compounds (Pedersen et al., 2005).The activity of TRPA1 can be regulated by desensitization and sensitization of calcium ions. In contrast, repeated activation of TRPA1 by chemical stimulation can lead to calcium- and current-dependent desensitization. TRPA1 has a clear function in the neuron system (Bandell et al., 2004; Bautista et al., 2006).TRPA1 not only plays an important role in the nervous system but also affects the control of local tissue inflammation and the progression of tissue fibrosis, which has become a research topic of great interest (Inoue et al., 2019).

TRPA1 is highly expressed in fibroblasts and has an important influence on many fibrotic diseases and pathological processes of cancers (Yin et al., 2018).As TRPA1 is a typical stress-responsive calcium/sodium channel, calcium channels can play a role in cell fibrosis through a series of signaling pathways, and a large amount of calcium influx can also lead to cell apoptosis. A high level of apoptosis and necrosis can promote the proliferation of fibroblasts and then lead to the corresponding fibrosis (Hasan and Zhang, 2018).At the same time, the upstream pathway of TRPA1 is also related to the proliferation of fibroblasts (Viana, 2016).It should be noted that a variety of allosteric coupling mechanisms *in vivo* can affect the activity of TRP channels (including TRPA1). The TRPA1 domain contains many protein-interacting sequences and phosphorylation sites. These sites respond to time-varying changes in the extracellular and intracellular environment (Mulier et al., 2017; Fliniaux et al., 2018).

Tissue fibrosis is a complex process that is affected by many factors. Among them, fibroblasts are the key factor in tissue fibrosis. Fibroblasts have obvious heterogeneity and diversity. When organs and tissues undergo physiological, pathological or immune reactions, fibroblasts will change accordingly. When fibrosis occurs, fibroblasts synthesize a large amount of extracellular matrix (ECM). Fibroblasts interfere with scar formation and organ lesions in fibrosis through the ECM (Lowin et al., 2015).

In the process of fibrosis, fibroblasts differentiate into myofibroblasts, which have proliferation and contraction functions. In pathological fibrosis, myofibroblasts persist in tissues and cause fibrosis through increased matrix synthesis and tissue contraction (Darby and Hewitson, 2007).It has been reported that TRPA1 has a certain effect on the differentiation, proliferation, extracellular matrix synthesis and survival of myofibroblasts (Li et al., 2019).Fibroblasts play a more complex role in liver fibrosis. It regulates the fibrosis process through the ECM. Hepatic stellate cells (HSCs) are crucial in hepatic fibrosis and pseudolobuli formation (Higashi et al., 2017).Cancer-associated fibroblasts (CAFs) act as a major cause in the formation of the tumor microenvironment (TME) (Yang et al., 2012).

In this brief review, we discuss the possible relationship between TRPA1 and fibroblasts in fibrosis-related diseases. Since the antifibrotic mechanism of TRPA1 is still unclear, we will start with fibroblasts and demonstrate the relationship between TRPA1 and the specific mechanism of fibrosis through TRPA1-related research (Okada et al., 2014). In addition, antifibrotic therapy related to TRPA1 has been confirmed by many studies. It is hoped that this review will provide a summary of the relationship of TRPA1 with fibrosis and discuss future research in this area (Tonello et al., 2017; Xu et al., 2019).

## Transient receptor potential ankyrin 1 structure and function in fibrosis

### Structure of transient receptor potential ankyrin 1

TRPA1 is a nonselective cation channel that is expressed on the cell membrane. TRPA1 channels can allow passage of sodium ions, calcium ions, and zinc ions. The TRPA1 ion channel is widely expressed in organisms and found to be enriched in mammalian peripheral receptive neurons. It participates in the sensory transduction of chemical and thermal stimuli and is involved in the process of inflammation and pain (Gouin et al., 2017).

**FIGURE 1:**
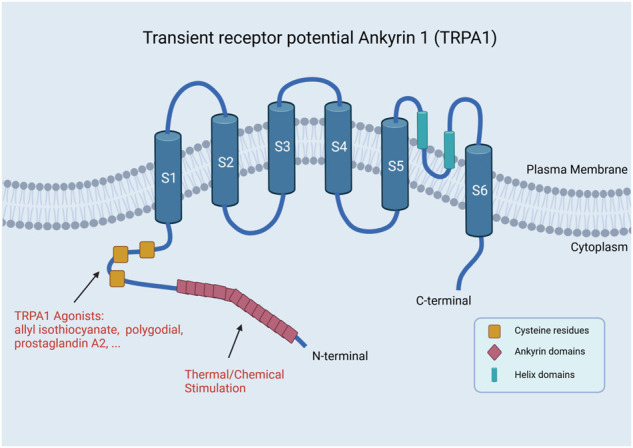
Structure of TRPA1. TRPA1 has a long N-terminal ankyrin domain, which is composed of multiple ankyrin repeats, mainly including the regions responsible for the interaction with other proteins and reception of external stimuli by the channel, such as thermal and chemical stimulation (Created with BioRender.com).

TPRA1 is typically a stress-responsive cation channel permeable to Ca^2+^ and other ions in which the N-terminal domain has 14 ankyrin repeats, while the other subfamilies have only three or four repeats (Figure 1) (Paulsen et al., 2015). One study has shown that 80% of the molecular weight of the TRPA1 subunit is in the N-terminal domain (Nazıroğlu and Braidy, 2017). The most characterized feature of TRPA1 is a long N-terminal ankyrin domain, which has the longest ankyrin repeat sequence in the TRP superfamily. The ankyrin domain contains regions that are responsible for the interaction with other proteins and for reception of external stimuli of the channel, such as thermal and chemical stimulation (Wilson et al., 2011; Chen and Hackos, 2015).

TRPA1 can also be stimulated by reactive oxygen species to activate calcium influx. The activity of TRPA1 is regulated by O_2_. The stimulation of hyperoxia is mediated by cysteine residues C633 and C856s glutathione-sensitive oxidation in the N-terminal domain of the channel (Takahashi et al., 2011).At the same time, reactive oxygen species, such as peroxides, can cause cysteine oxidation in the human body. Cysteine oxidants can activate TRPA1 (Sawada et al., 2008). Oxidative stress can produce not only reactive oxygen species but also oxidized phospholipids (OXPAPC). OXPAPC can activate TRPA1 by modifying cysteines, causing acute pain and hyperalgesia and releasing preinduced pain peptide (Liu et al., 2016; Oehler et al., 2017).

In electrophysiological analyses, TRPA1 has been shown to be a sensitive and low-threshold electrophoretic receptor. In mammals, pain and neurogenic inflammation are induced by the TRPA1 subtype (named after its characterized ankyrin repeat domain), which is then expressed by primary afferent nociceptors through toxic compounds (Chen et al., 2013). Selective TRPA1 antagonists have been investigated for years and have been proven beneficial, but their site and mechanism of action are still unclear. In 2007, HC–030031, developed by Hydra Biosciences, was the first TRPA1 antagonist (McNamara et al., 2007). Many TRPA1 agonists activate the channels by covalently modifying the conserved cysteine or lysine residues in the cytoplasm (Hinman et al., 2006; Macpherson et al., 2007).

There are many special structures in TRPA1, such as helix domains, which have special mechanisms. TRPA1 interacts with Inositol hexakisphosphate (InsP6) by stabilizing the helix domain. When phospholipase-C-coupled receptor hydrolyzes phosphatidylinositol-4,5-diphosphate to produce inositol polyphosphate, polyphosphate acts as the second messenger to regulate TRPA1 activity together with cytoplasmic calcium and G proteins. There are many similarities between TRPA1 and TRPV1. These two channels are assembled into homotetramers, which can exchange domains at the transmembrane core. One of the channels is regulated by the ion permeation pathway controlled by two restriction points, and the other one is regulated by a conserved isoleucine residue. The interaction between these cytoplasmic subunits can regulate channel assembly and/or promote cooperative conformational changes. This is similar to cofactor binding or agonist-induced transmembrane core domain exchange (Cao et al., 2013; Liao et al., 2013). This leads to a similar function of TRPA1 and TRPV1 in fibroblasts.

### Function of transient receptor potential ankyrin 1 in fibrosis

**FIGURE 2:**
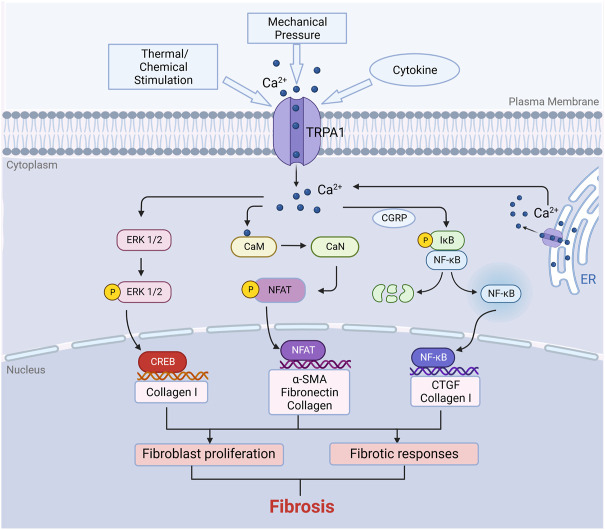
Function of TRPA1 in fibrosis. TRPA1 can be stimulated by temperature, chemicals, mechanical forces and cytokines such as IL-6, TNF-α and TGF-β. Activation of TRPA1 protein induces calcium influx in fibroblasts. Calcium influx activates several signaling pathways. The increase in calcium influx in fibroblasts leads to the binding of calmodulin to calcium ions, which further activates calcineurin. Calcineurin induces the dephosphorylation of NFAT in the cytoplasm, and then the dephosphorylated NFAT enters the nucleus to regulate gene expression, resulting in cell proliferation and immune responses. Similarly, the increase in intracellular calcium can also activate the ERK/CREB and NF-κB signaling pathways to promote cell proliferation. Fibroblast proliferation consequently produces ECM, which results in fibrosis (Created with BioRender.com).

Why did we focus on the role of TRPA1 in fibrosis? The main reason is that the calcium ion signaling pathway shows a relationship with inflammation and apoptosis. The inflammatory response is the premise of many signaling pathways in cell fibrosis. Apoptosis is an important process that leads to changes in cells after stress that can result in fibrosis. Many studies on TRP family-related fibrosis have been carried out. The TRP family is closely related to myocardial fibrosis (Inoue et al., 2019). In the TRP family, TRPV4 and TRPA1 often show high expression in fibroblasts at the same time. However, many studies have only focused on the relationship between TRPV4 and fibrosis, and few have discussed TRPA1. Therefore, we hope to review the specific relationship between TRPA1 and fibrosis through existing studies (Figure 2). (Zhan and Li, 2018)

Tissue fibrosis is usually associated with tissue damage. Injury causes local inflammation and promotes tissue repair. Fibrosis is an important process for repairing tissue. However, excessive inflammation and subsequent tissue fibrosis/scarring may impair the physiological function of tissues and organs. Due to its characteristic calcium channel activity, the activation of TRPA1 leads to increased calcium influx, decreased proliferation and increased necrosis. The inflammatory tissue fibrosis process involves many growth factors and cytokines. However, transforming growth factor-β (TGF-β) is closely related to TRPA1-related fibrosis (Stover et al., 2007).Some experiments have shown that the loss of TRPA1 signaling inhibits corneal inflammation and fibrosis after chemical injury. In fibroblasts, inhibition of the TGF-β1-related signaling cascade may be involved in the mechanism of TRPA1-related fibrosis (Okada et al., 2014).

TRPA1 is required for TGF-β signaling transduction (Stover et al., 2007). TGF-β is a multifunctional fibrotic remodeling mediator that induces myofibroblast transformation, Smad activation, and ECM production. TGF-β signaling is transduced primarily through TGF-β receptor (TβR)-mediated Smad and non-Smad signaling. Activated TGF-β binds to TGF-β receptor 2 (TGFβR2) and activates TGF-β receptor 1 (TGFβR1), resulting in the phosphorylation of Smad 2 and Smad 3. p-Smad 2/3 and Smad 4 form Smad complexes, which are then transported to the nucleus to regulate collagen, fibronectin and α-smooth muscle actin (α-SMA) and finally form tissue fibrosis (Meng et al., 2016).

Under inflammatory conditions, TGF-β upregulates the expression of TRPA1. Mitogen-activated protein kinase (MAPK) and other kinases, including c-Jun N-terminal kinase (JNK) and p38 MAPK, are activated by TGF-β (Massague, 2003). TGF-β1 promotes fibrosis by inducing resting fibroblasts to differentiate into myofibroblasts that secrete matrix (Verrecchia et al., 2001). Reactive oxygen species (ROS) produced by inflammation induced by the TGF-β signaling pathway can activate TRPA1 and produce corresponding pathophysiological effects in fibroblasts through calcium influx (Yin et al., 2020). It is worth noting that the ROS/TRPA1/CGRP signaling pathway can promote cell proliferation and differentiation in fibroblasts (Li et al., 2020).

Activation of the TRPA1 channel leads to calcium influx. Calcium ions are closely related to the regulation of fibroblast gene expression (Janssen et al., 2015). By activating fibroblasts, intracellular isolated Ca^2+^ pulse release is triggered, and then membrane conduction is activated, including the voltage-dependent Ca^2+^ influx pathway. TRPA1 is one of the downstream proteins of the inflammatory response. In general, these events produce repetitive calcium oscillations, which are mediated by calcium-dependent transcription factors and regulate various cellular physiological processes. Cell proliferation, extracellular matrix protein synthesis/secretion, self-activation (TGF-β production) and transformation into myofibroblasts are all related to this process (Pozsgai et al., 2010).

A variety of inflammatory factors can also activate TRPA1 by producing ROS. The expression of IL-6 was upregulated by TRPA1. Moreover, IL-6 can promote the fibrotic function of TRPA by upregulating inflammation (Engler et al., 2007). TNF-α can act as an inflammatory factor upstream of TRPA1 and induce fibroblast proliferation. At the same time, TNF-α can induce fibroblast apoptosis by mediating TRPA1-induced calcium overload, which further aggravates fibrosis and organ dysfunction (Kameda et al., 2019).

The increase in calcium ions in fibroblasts leads to the binding of calmodulin. The complex activates calcineurin to dephosphorylate NFAT. After dephosphorylation, NFAT enters the nucleus and regulates genes to upregulate cell proliferation and induce immune responses (Wang et al., 2016a). Calcium can activate the MAPK pathway in fibroblasts (Uwada et al., 2019). Additionally, another experiment showed that the ERK/CREB signaling pathway makes an important contribution to fibroblast proliferation.

Previous studies have shown that fibroblast populations do exhibit mutations independent of epithelial defects in human cancers. These mutations can affect important regulatory pathways, leading to the activation of matrix components, thus promoting the occurrence and development of cancer (Affo et al., 2017). Other studies confirmed the possible relationship between TRPA1 and fibrosis-related cytokines. FGFR2 and TRPA1 form a complex. The binding mode is that the ankyrin repeat of TRPA1 binds to the proline-rich region of FGFR2. This interaction has special physiological significance and may inhibit the activity of TRPA1 through structural changes in the TRPA-1 conformation. As a possible conformational scaffold, this structural change can induce receptor activation without extracellular stimulation, leading to abnormal proliferation and invasion (Berrout et al., 2017).

## Modulation of fibrosis by transient receptor potential ankyrin 1 in multiple diseases

**FIGURE 3:**
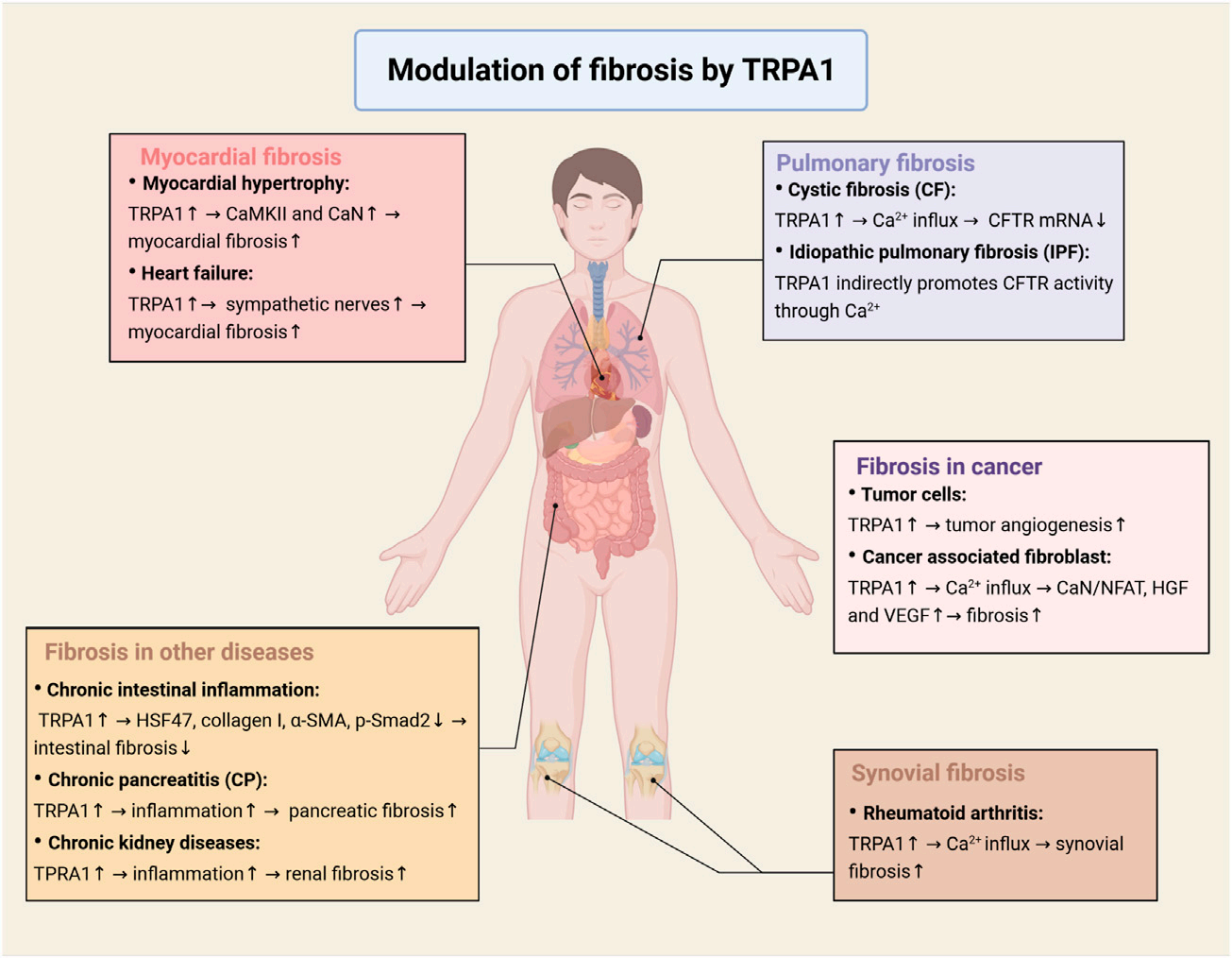
Modulation of fibrosis by TRPA1 in multiple diseases. In myocardial diseases, TRPA1 regulates the activation of the CaMKII and calcineurin signaling pathways in hypertrophic cardiomyocytes and leads to an increase in sympathetic nerves in heart failure, which promotes myocardial fibrosis and fibroblast differentiation. In synovial diseases, activation of TRPA1 causes calcium influx, which promotes synovial fibroblast proliferation. In pulmonary diseases, TRPA1 regulates the expression of CFTR protein through calcium, which is responsible for cystic fibrosis and idiopathic pulmonary fibrosis. In cancer, TRPA1 plays an important role in tumor angiogenesis, which activates neovascularization *in vivo* and increases cell migration and tubule formation *in vitro*. Calcium influx induced by TRPA1 activation will activate the CaN/NFAT signaling pathway and secrete HGF and VEGF to promote cell proliferation. Apart from the diseases mentioned above, TRPA1 affects fibrosis in other diseases, such as intestinal fibrosis, pancreatic fibrosis and renal fibrosis (Created with BioRender.com).

In the last section, we described that the activation of TRPA1 promotes fibrosis, but we did not discuss how this occurs in specific diseases. In this section, we will review the regulatory role of TRPA1 in fibrosis in myocardial diseases, synovial diseases, pulmonary diseases, cancer and other diseases (Figure 3).

### Myocardial diseases

Myocardial fibrosis is the pathological change in the myocardium after myocardial injury or overcompensation, which is the mechanism of myocardial healing. As pathological compensation, myocardial fibrosis is usually considered beneficial in the early stage, but excessive fibrosis leads to irreversible damage. In the experiment, we found that the expression of α-SMA, collagen and ECM increased in the myocardium (Kong et al., 2014). It is more important that fibroblasts play a leading role in myocardial fibrosis (Shinde and Frangogiannis, 2014).

The calcium regulatory signaling pathway has been proven to be responsible for pathological myocardial hypertrophy and related gene activation. Myocardial hypertrophy is a disease mainly caused by myocardial fibrosis. Further studies have shown that CaMKII and calcineurin, the calcium-dependent signaling pathway, are considered signal effectors of central hypertrophy in the myocardium (Wilkins and Molkentin, 2004; Kreusser et al., 2014). Some studies have shown that inhibition of TRPA1 reduces myocardial fibrosis induced by pressure overload (Wang et al., 2018).

TRPA1 is highly expressed in cardiac fibroblasts and acts as a leading factor in the increase in intracellular calcium. Some studies have shown that calcium ions promote the proliferation, differentiation and ECM formation of fibroblasts. There are several signaling pathways in cardiac fibroblasts that are influenced by calcium ions. The increasing amount of calcium ions tends to bind to calmodulin (CaM). This binding leads to the activation of calcineurin (CaN). Furthermore, CaN has the ability to phosphorylate proteins. After the NFAT protein is phosphorylated by CaN, it can upregulate target genes to promote cell proliferation and differentiation. Furthermore, the CaN/NFAT signaling pathway influences the immune response of cardiac fibroblasts.

Another important signaling pathway is the ROS/TRPA1/CGRP signaling pathway. The activation of the MAPK signaling pathway and CaMKII-STAT3 signaling pathway is of great importance in the secretion and proliferation of cardiac fibroblasts and the promotion of myocardial fibrosis (Kumar et al., 2019). Experiments have confirmed that calcium inflow caused by stored-operated calcium entry (SOCE) can cause p38 MAPK phosphorylation (Uwada et al., 2019). It has been proven that calcium can affect the MAPK pathway in fibroblasts. CGRP is produced by autocrine secretion by cardiac fibroblasts. Autocrine CGRP can attenuate fibrosis by promoting the NF-κB signaling pathway. In a model of myocardial hypertrophy and fibrosis, the NF-κB signaling pathway promotes the proliferation and differentiation of cardiac fibroblasts (Higashikuni et al., 2013; Das et al., 2018). The NF-κB signaling pathway may promote the process of fibrosis by upregulating type I collagen and CTGF genes (Thakur et al., 2014).

In conclusion, TRPA1 regulates the activation of the CaMKII and calcineurin signaling pathways in hypertrophic cardiomyocytes. In addition, TRPA1 can result in an increase in sympathetic nerves in a heart failure model (Adam et al., 2019). Sympathetic nerve stimulation promotes myocardial fibrosis and fibroblast differentiation, which aggravates cardiac failure. These results suggest that TRPA1 plays a crucial role in the different pathological processes of myocardial fibrosis and is of great significance in the follow-up process of myocardial lesions. TRPA1 is a possible therapeutic intervention target in the clinic.

### Synovial diseases

Normal synovial tissue consists of synovial fibroblasts (SFs) and macrophages. Studies of TRPA1 suggest that TRPA1 can effectively reduce the inflammatory response of SFs. Studies have shown that TRPA1 is related to the production of inflammatory mediators. TRPA1 also plays a key role in severe arthritis (Lowin et al., 2015). In rheumatoid arthritis synovial fibroblasts, the expression of TRPV1 and TRPA1 and the regulation of related receptors by endogenous agonists have been shown to reduce the production of IL-6, IL-8 and MMP-3 (Engler et al., 2007). Results have shown that pain sensitivity is decreased in TRPA1 gene knockout mice, which indicated that TRPA1 could increase calcium flow in isolated synovial cells (Kochukov et al., 2006; Horváth et al., 2016).

Synovial fibroblasts are affected by rheumatoid arthritis, and the cytokine TNF upregulates TRPA1, the activation of which can increase the intracellular calcium level, leading to lactate dehydrogenase release. LDH is positively related to cell necrosis and pathological changes. Among them, the changes in calcium in cells are related to the proliferation and survival of fibroblasts. Rheumatoid arthritis synovial fibroblasts show a strong ability to proliferate and inhibit apoptosis *in vivo*. IL-6 and IL-8 are the major inflammatory regulators in rheumatoid arthritis. At the same time, TRPA1 agonists have also been shown to reduce the production of IL-6 and IL-8 (Lowin et al., 2018).

TRPA1 is considered to be a potential target for relieving arthritis pain. However, evidence has been provided that TRPA1 may be mainly located in the inflammatory areas of joints that are rich in TNF and may also directly affect RA effector cell functions (Bertin et al., 2017). SFs are a potential therapeutic target because they produce inflammatory mediators and cause tissue destruction. Moreover, SFs induce the production of autoreactive B lymphocytes and T lymphocytes in tissues and joint destruction through the production of proteases (Soutar et al., 2017). Therefore, a decrease in SFs in synovial tissue will reduce the pressure of inflammation and other proinflammatory cells. In conclusion, TRPA1 is a potential receptor for reducing the number of SFs (Anand et al., 2008). Emerging data suggest that inhibition of TRPA1 can effectively reduce the inflammatory response of SFs.

### Pulmonary diseases

In pulmonary inflammatory diseases, pulmonary fibroblasts are the key factors of pulmonary fibrosis. Our objective was to investigate the functional expression of TRPA1 in a human alveolar epithelial cell line (A549) and lung fibroblasts (ccd19-Lu). Allyl isothiocyanate (AITC) has an excitatory effect on it, while TRPA1 can also induce fibroblasts to secrete inflammatory factors such as IL-8 (Mukhopadhyay et al., 2011). Furthermore, TRPA1 activation is associated not only with the increased release of IL-8 cytokines but also with the upregulation of matrix metalloproteinase 9 (MMP9) gene expression in human lung fibroblasts treated with TNF-α (Yap et al., 2020).

However, the relationship between TRPA1 and lung fibroblasts is complex. It has been reported that AITC can promote α–SMA induced by TGF-β1 by activating the ERK1/2 MAPK and Nrf2/HO-1 pathways in lung fibroblasts. This also overcomes the problem that α-SMA induced by TGF-β1 is not sensitive to corticosteroids. TRPA1 antagonist modulates the inhibition but does not prevent it. AITC and TRPA1 antagonists may be therapeutic agents for chronic respiratory diseases (Yap et al., 2021).

Similarly, many lung diseases are characterized by fibrosis. These processes of fibrosis are closely related to fibroblasts. Cystic fibrosis (CF) is generally considered to be a genetic disease caused by mutations in the CF transmembrane conduction regulator (CFTR) gene (Meng et al., 2017). CFTR is a protein belonging to the ATP binding cassette superfamily. It is characterized by its ability to allow special ions to pass through the apical plasma membrane (PM) of epithelial tissue (including the airway) (Antigny et al., 2011). The expression of proinflammatory mRNAs and proteins in CF is higher than that in wild-type fibroblasts, and the latter is similar to that in other nonepithelial cells (Huaux et al., 2013).

CFTR is a channel regulated by cAMP and ATP to ensure the transport of Cl^−^ and bicarbonate (Ribeiro et al., 2005). Ca^2+^ homeostasis is of great importance in the physiology of CF cells. To our knowledge, the expression of CFTR protein is affected by the increase in intracellular calcium, which downregulates the level of CFTR mRNA. In addition, the increase in intracellular calcium is related to changes in immune and respiratory responses (Patel et al., 2019). TRPA1, as a major calcium regulatory protein in fibroblasts, may act as a major regulator in the regulation of CFTR.

The literature related to TRPA1 described the regulation of the inflammatory response by the TRPA1 channel in CF bronchial epithelium induced by bacterioplankton supernatant or mucopurulent substances. One study demonstrated that TRPA1’s selective antagonists inhibited TRPA1 calcium transport and gene silencing, which resulted in an obvious decrease in related protein expression, significantly reducing the transcription process and inhibiting the release of different inflammatory cytokines. It is suggested that TRPA1 may be helpful for improving CF and has therapeutic significance for pulmonary fibroblast-associated inflammation (Prandini et al., 2016). At the same time, these authors also published a paper confirming that the use of agonists directly activating TRPA1 can induce small airway epithelial cells to release IL-8 (Nassini et al., 2012).

Idiopathic pulmonary fibrosis (IPF) is a chronic progressive pulmonary disease with unknown etiology. The most common symptoms are cough, dyspnea and fatigue. The TRPA1 channel helps to transfer extracellular signals into cells and indirectly promotes CFTR activity through Ca^2+^. The TRPA1 channel is expressed in various cells in the respiratory mucosa, including sensory nerves, lymphocytes, epithelial cells, smooth muscle cells and fibroblasts (Jaquemar et al., 1999). The upregulation of TRPV1/TRPA1 expression in bleomycin-induced chronic cough in guinea pigs provides a new idea for the mechanism of IPF-related cough allergy (Guo et al., 2019).

### Other fibrotic diseases

Intestinal fibrosis is a common complication of inflammatory bowel disease (IBD), including ulcerative colitis (UC) and Crohn’s disease (CD) (Bettenworth and Rieder, 2014). Ulcerative colitis (UC) lesions are limited to the epithelial mucosa and submucosa, the pathological manifestations are moderate, and CD lesions across the entire intestinal and colon wall are recurrent and progressive. Approximately one in three CD patients has severe intestinal stenosis and obstruction because of recurrent local chronic inflammation. This late fibrosis state is considered irreversible.

Chronic intestinal inflammation is an important inducer of intestinal fibrosis (Rieder et al., 2007). Fibroblasts are of great significance in the process of fibrosis (Valentich et al., 1997). In the process of intestinal fibrosis, mechanical stretching and anti-inflammatory ligands (e.g., TGF-β) cause fibroblast transformation. In fibroblasts, it has been proven that increased expression of constitutive N-cadherin can enhance the formation of stenosis in CD patients (Burke et al., 2011). TGF-β acts as a vital factor in the pathogenesis of CD fibrosis and stenosis (Biancheri et al., 2014). In inflammatory bowel disease patients, the level of TGF-β in the inflammatory bowel increases, and atypical TGF-β signaling impairs immune tolerance and tissue repair in the intestine (Babyatsky et al., 1996).

In Crohn’s disease-related intestinal fibrosis, activation of TRPA1 on fibroblasts alleviates fibrosis. Steroid- and pirfenidone-induced TRPA1 activation antagonizes TGF-β1-induced expression of heat shock protein 47, type 1 collagen and α-smooth muscle actin and decreases Smad-2 phosphorylation and myocardial protein expression. The expression of TRPA1 was significantly increased in the narrow intestinal region of CD patients (Kurahara et al., 2018). Daikenchuto (Da-Jian-Zhong-Tang, DKT) can inhibit intestinal fibrosis by activating TRPA1 channel expression. Moreover, DKT inhibits the expression of α-smooth muscle actin (α-SMA) and type I collagen induced by TGF-β1, with p38 mitogen activated protein kinase (p38 MAPK) and Smad-2 being phosphorylated and myocardial protein expression being decreased (Hiraishi et al., 2018).

Chronic pancreatitis (CP), along with partial or total loss of function, is a debilitating disease characterized by inflammation and irreversible morphological changes. Pancreatic acinar atrophy, pancreatic duct deformation, fibrosis and calcification are observed to different degrees, and pancreatic exocrine and endocrine dysfunctions have also been observed. Pancreatic fibrosis is the result of pancreatic injury and repair (Dimcevski et al., 2007).

TRP channel antagonists can reduce pancreatitis, improve behaviors related to pain and prevent the progression of pancreatic histopathological changes and the upregulation of TRPA1, TRPV1, and PERK in pancreatic afferent fibers. Fibrosis and sprouting of pancreatic nerve fibers have been observed in CP. PERK, TRPA1 gene transcription and noxious markers are significantly increased. Neurogenic inflammation plays a key role in pancreatitis and pain-related behaviors. Early intervention with TRP channel antagonists can effectively inhibit the transformation and development of CP (Schwartz et al., 2013).

Renal fibrosis mainly refers to renal interstitial fibrosis. Some chronic kidney diseases, as well as interstitial nephritis or chronic nephritis and other diseases, will cause structural and morphological changes in the kidney, renal interstitial fibrosis, and glomerulosclerosis and eventually lead to an increase in creatinine and urea (Horowitz et al., 2015).

Acute tubular necrosis (ATN) is the most common cause of acute tubular necrosis (AKI), including tubular cell injury and death. Oxidative stress has a great influence on the pathophysiology of ATN (Pavlakou et al., 2017). After renal injury, oxidative stress, characterized by an increase in reactive oxygen species (ROS) and/or reactive nitrogen species, can induce complex mechanisms of tubular injury directly or indirectly. TRPA1 can be activated by toxic or inflammatory mediators (such as ROS), and activation of TRPA1 may aggravate the inflammatory response and lead to renal fibrosis (Gorin, 2016). The results showed that the high expression of TRPA1 in renal tubules was related to the recovery of renal function after renal injury (Wu et al., 2019).

TRPA1 deficiency decreases the expression of IL-6, TGF-β1 and α-smooth muscle actin but inhibits the activation of p38, Smad3, MAPK, ERK and JNK. In fibroblasts, TGF-β1 signaling pathway inhibition induced by loss or blockade of TRPA1 can reduce corneal stromal inflammation and fibrosis/scarring (Okada et al., 2014).

### Cancers

The TGF-β signaling pathway plays an upstream and downstream regulatory role in TRPA1 fibrosis (Feng and Derynck, 1996). Activated TGF-β has been recognized to regulate spontaneous epithelial cell tumorigenesis, development and metastasis. It has been reported that the interaction between cancer cells and the local tumor microenvironment is mediated by TGF-β. Epithelial cell-independent TGF-β signal transduction regulates the tumorigenesis and development of mouse and human tumors. Similarly, interactions in the TME, such as fibroblast recruitment/activation, epithelial fibroblast-related crosstalk, and ECM modification, are potentially regulated by TGF-β, which eventually promotes cancer progression. Tumor vessels in epithelial fibroblasts play an important role in tumorigenesis and development (Harpel et al., 2001).

During the growth of tumor cells, TRPA1 acts as a critical mediator in tumor angiogenesis. In addition, we have demonstrated that TRPA1 is a pivotal positive regulator of vascular morphogenesis, which activates neovascularization *in vivo* and epithelial cell migration and tubulogenesis *in vitro*. We found that the wound healing of prostate tumor-derived endothelial cells (PTECs) is increased by endogenous TRPA1 activation, which can be inhibited by siRNA transfection of the downregulation channel. Consistent with these results, cell migration and tubule formation of human microvascular endothelial cells (HMECs) *in vitro* is increased due to overexpression of TRPA1 (Bernardini et al., 2019). The literature suggests that resveratrol may have agonistic and antagonistic effects on TRPA1 in different species. Resveratrol**-**activated human TRPA1 can induce the entry of Ca^2+^ and lead to the expression of growth factors, which may regulate growth and migration and inhibit apoptosis in cancer-associated fibroblasts (CAFs) through secretion of HGF and VEGF and the calcineurin/NFAT pathway, which should be further researched for confirmation (Vancauwenberghe et al., 2017).

Based on the present research, we can conclude that TRPA1 in fibroblasts is of great significance in the process of fibrosis and has good prospects as a target in the treatment of fibrosis-related diseases. However, until now, few studies have reported the relationship of TRPA1 and cancer-associated fibroblasts in fibrosis, which remains a mystery for scientists to reveal.

## Transient receptor potential ankyrin 1 as a potential target in antifibrotic therapy

It is indisputable that TRPA1 may be used to treat fibrosis. However, its application in the clinic has been difficult. Here, we review the agonists and antagonists of TRPA1 (Logashina et al., 2019).

The TRPA1 channel may interact with many compounds. The mechanism of TRPA1 activation can be divided into two types: electrophilic and nonelectrophilic. Electrophilic agonists bind to TRPA1 by modifying amino acid residues. At present, it is believed that electrophilic TRPA1 agonists are mainly in the form of covalent bonds. Among these, the most notable finding is that the TRPA1 amino acids that electrophilic agonists bind to are mainly cysteine and lysine residues. The mutation or transformation of the structure may have a great influence on its effect.

It should be noted that calcium is the best enhancer for agonists. Intracellular calcium can directly activate TRPA1 and significantly positively regulates its existing excitatory effect. The calcium ion interaction between calmodulin and the calmodulin binding domain of TRPA1 can further regulate the sensitivity of TRPA1 to calcium ions (Macpherson et al., 2007).

Compared with TRPA1 agonists, the number of antagonists of TRPA1 is smaller, and the effect is not as good as agonists. The current research is very limited. In IC50 assessments, some TRPA1 antagonists have been shown to require TRPA1 activation induced by TRPA1 agonists, yet there are a number of TRPA1 antagonists that can act directly on TRPA1. The sources of antagonists are mainly natural and synthetic. It has been proven that a nontoxic compound known as cardamonin, which is isolated from the Zingiberaceae herb, can selectively inhibit TRPA1 (IC50 = 454 nM) and bind to the A967079 antagonist (Takaishi et al., 2012). Due to the scarcity of natural inhibitors of TRPA1, there are many studies utilizing synthetic and selective antagonists. At present, approximately 50 kinds of compounds have been proven to be effective. Xanthine derivative HC030031 represents the first generation and was the earliest to be synthesized (Wang et al., 2016b). It acts on the channel at a micromolar concentration. However, due to its poor solubility, it cannot be used in clinical trials. A967079 is one of the most commonly used compounds in the laboratory. Its role in pain treatment and the inflammatory response has been confirmed. Research on its anti-fibrotic activity is still in progress (Sałat et al., 2013). In a phase 1 study with healthy volunteers, dermal blood flow, pain, and itch induced by TRPA1 agonists were shown to be reduced by treatment with GDC-0334, a highly potent, selective, and orally bioavailable TRPA1 antagonist, suggesting that GDC-0334 can be a potential therapeutic for humans (Balestrini et al., 2021) (Table 1).

**TABLE 1:** TRPA1 antagonists.

Compound	Selectivity	IC_50_	Clinical use	References
Cardamonin	Yes	454 nM		Takaishi et al. (2012)
A967079	Yes	Human:67 nM	Yes	WO2009089082 (2009)
		Rat:289 nM		
HC030031	Yes	4.9 μM	Yes	WO2007073505 (2007)
		7.5 μM		
CB-625			Phase I completed	
GRC-17536	Yes	<10 nM	Phase IIa clinical trial (NCT01726413)	
GDC-0334	Yes	Human: 1.7 nM	Yes	Balestrini et al. (2021)
		Mouse:2.7 nM		
BI01305834	No	0.05 μM		

## Conclusion

As a calcium channel protein, TRPA1 regulates the excitability of pain-sensing neurons. In many pain treatments, TRPA1 has undoubtedly become an important target. In contrast, the role of TRPA1 in the progression of fibrosis is unknown. Many experiments have shown that TRPA1 is highly expressed in fibroblasts of specific organs. The function of TRPA1 is involved in many stages of fibrosis and may play an indispensable role in the process of fibrosis.

TRPA1 can be stimulated by cytokines in fibroblasts to promote the proliferation of fibroblasts. However, TRPA1 may play different roles in the process of fibrosis and may induce fibroblast differentiation. We believe that summarizing the different mechanisms of TRPA1 and the relationship between TRPA1 and the cell pathways can be helpful in understanding the mechanism by which fibroblasts are regulated.

In conclusion, TRPA1 may be a potential target in inflammation and inflammation-related fibrosis. In some advanced stages of fibrosis, TRPA1 antagonists can be used as an adjuvant therapy. It is worth mentioning that TRPA1 is a calcium channel that can antagonize fibrosis and relieve inflammation and pain. This is an advantage in antifibrotic therapy.

TRPA1 is highly expressed in tumor tissues, especially in tumor fibroblasts. This may become a direction in future research on fibroblast-related cancer.
